# Life-Threatening Gastrointestinal Bleeding in a Child with Acute Hepatitis A

**DOI:** 10.3390/children13040526

**Published:** 2026-04-10

**Authors:** Simona Zlatanova, Meri Hristamyan, Kostadin Ketev

**Affiliations:** 1 Department of Infectious Diseases, Parasitology and Tropical Medicine, Faculty of Medicine, Medical University of Plovdiv, Blvd Vasil Aprilov 15A, 4002 Plovdiv, Bulgaria; 2 Clinic of Infectious Diseases and Parasitology, St. George’s University Hospital, 4002 Plovdiv, Bulgaria; 3 Department of Epidemiology and Disaster Medicine, Faculty of Public Health, Medical University of Plovdiv, 4002 Plovdiv, Bulgaria; meri.hristamyan@mu-plovdiv.bg; 4 Department of Pediatrics, St. George’s University Hospital, 4002 Plovdiv, Bulgaria; kostadin.ketev@mu-plovdiv.bg; 5 Medical Simulation Training Center, Medical University of Plovdiv, 4002 Plovdiv, Bulgaria

**Keywords:** hepatitis A, pediatrics, gastrointestinal bleeding, duodenal ulcer, hyperbilirubinemia, coagulopathy

## Abstract

Background: Hepatitis A virus (HAV) infection is a common cause of acute viral hepatitis in children. Severe complications are rare but may occur, particularly in older children or in the presence of concomitant conditions. Case Presentation: We report the case of an 11-year-old girl with acute hepatitis A with severe hepatic derangements who developed life-threatening upper gastrointestinal bleeding due to a previously undiagnosed duodenal ulcer. Emergency endoscopy confirmed active bleeding from a duodenal ulcer, and the patient survived the complications with treatment with a proton pump inhibitor and hemostatic management with blood products. Conclusions: Although hepatitis A is generally benign in children, this case highlights the potential for severe and life-threatening complications.

## 1. Introduction

Hepatitis A virus (HAV) infection remains a significant global public health concern and represents one of the leading causes of acute viral hepatitis worldwide [1]. Although HAV infection does not progress to chronic disease and is typically self-limiting, its transmission remains closely associated with inadequate sanitation, limited access to clean water, and insufficient vaccination coverage [1].

In Europe, hepatitis A is characterized by low endemicity, although certain high-risk populations—including men who have sex with men, travelers to endemic regions, and unvaccinated individuals—remain vulnerable to infection and outbreaks [2]. Bulgaria is considered a low- to intermediate-endemicity country, with seroepidemiological studies conducted between 2000 and 2013 demonstrating a seroprevalence of approximately 22% by the age of 30 and over 70% in adults older than 40 years, reflecting a shift in infection toward older age groups as sanitation conditions improved [3,4,5].

HAV infection typically presents with an abrupt onset after an incubation period of 15 to 50 days (mean 28 days) and is transmitted primarily via the fecal–oral route through contaminated food or water or close interpersonal contact [1]. Clinical manifestations range from asymptomatic infection or mild prodromal symptoms—such as fatigue, nausea, vomiting, abdominal discomfort, and low-grade fever—to jaundice and hepatomegaly [1]. In rare cases, estimated at less than 1%, the disease may progress to fulminant hepatitis and acute liver failure [1].

In children younger than six years, HAV infection is frequently asymptomatic or mildly symptomatic, with jaundice occurring infrequently and spontaneous recovery typically achieved within two months [6]. In contrast, older children and adolescents more often develop clinically overt disease characterized by jaundice, pale stools, dark urine, and marked elevations in liver enzymes [6]. Fulminant hepatitis, although rare in pediatric populations, is more likely to occur in older children, during outbreaks, or in the presence of underlying comorbidities and may be accompanied by encephalopathy, coagulopathy, and life-threatening complications [1,7].

Vaccination remains the most effective strategy for preventing hepatitis A [1]. Vaccination is recommended for individuals at increased risk, including travelers to endemic regions. Despite targeted vaccination efforts, a significant proportion of children and adolescents remain susceptible to infection. Consequently, severe and atypical clinical presentations of HAV infection may still occur in unvaccinated pediatric patients, as illustrated by the present case [1,6].

## 2. Case Presentation

### 2.1. Clinical History

An 11-year-old girl was admitted to the Clinic of Infectious Diseases in Plovdiv with serologically confirmed acute hepatitis A virus (HAV) infection (anti-HAV IgM positive). Initial symptoms included nausea, vomiting, abdominal pain, dark urine, acholic stools, and scleral icterus. The patient had been previously hospitalized at a regional hospital for approximately 10 days, where the diagnosis of acute HAV infection was established. She was discharged after temporary clinical improvement, having received supportive therapy, dietary recommendations, and a plan for outpatient follow-up. Five days later, she was readmitted due to worsening symptoms, including persistent jaundice, abdominal discomfort, anorexia, vomiting, and dark urine. According to available medical documentation and parental report, the patient had not received corticosteroids, nonsteroidal anti-inflammatory drugs, or other ulcerogenic medications in the week preceding the episode of gastrointestinal bleeding.

### 2.2. Clinical Findings on Admission

On admission, the patient was in impaired general condition, afebrile, and hemodynamically stable. Physical examination revealed marked jaundice of the skin and dark yellow sclerae, consistent with cholestasis. Hepatomegaly was present, with the liver palpable approximately 5 cm below the right costal margin and with firm consistency on palpation. No splenomegaly was observed.

Laboratory parameters on admission are summarized in Table 1. Initial labs demonstrated moderate leukocytosis (16.17 × 10^9^/L), with an otherwise normal complete blood count.

### 2.3. Diagnostic Assessment

The diagnostic evaluation included a comprehensive clinical and epidemiological assessment; laboratory testing; and serological, immunological, and microbiological analyses, as well as imaging studies. Etiological diagnosis was established using an enzyme-linked immunosorbent assay, which revealed positive anti-HAV IgM antibodies, confirming acute infection. Serological markers for hepatitis B, C, and E viruses were negative, excluding co-infections.

Due to the severity and atypical progression of the disease, autoimmune liver disease was also investigated. A comprehensive autoimmune panel, including antinuclear antibodies, antimitochondrial antibodies, anti-smooth muscle antibodies, and other disease-specific antibodies, was performed, and all results were within normal reference ranges.

Other potential causes of acute liver injury, including viral co-infection, autoimmune liver disease, and metabolic disorders, were considered and excluded. Serum ammonia levels and Factor VII activity were not assessed during the acute phase of hospitalization, as these tests were not routinely available at the time of clinical evaluation.

### 2.4. Hospital Course

On the third day of hospitalization, the patient developed sudden-onset severe abdominal pain, accompanied by repeated vomiting containing hematin material and fresh blood, as well as melena. The clinical presentation, along with laboratory findings, suggested acute gastrointestinal bleeding, which was subsequently confirmed by upper gastrointestinal endoscopy.

The findings from upper gastrointestinal endoscopy revealed a stomach filled with fresh blood, hyperplasia of lymphoid follicles in the antrum and duodenum with a fine granular appearance of the mucosa, and the presence of an ulcerative lesion measuring approximately 15 mm in diameter on the posterior wall of the duodenal bulb, associated with active venous bleeding and adherent clot formation.

Throughout hospitalization, the patient was closely monitored, with supportive care for acute HAV infection, including hydration, rest, and liver function monitoring. Following the identification of a bleeding duodenal ulcer during endoscopic evaluation, proton pump inhibitor therapy was initiated as part of the conservative management.

Clinical chemistry investigations also demonstrated abnormalities, which are presented in Table 2.

Abnormalities in coagulation parameters were observed during hospitalization, as presented in Table 3. On admission, a reduced prothrombin index (PT%), corresponding to prolonged prothrombin time, together with mildly elevated international normalized ratio (INR), indicated impaired hepatic synthetic function. Despite transient improvement, persistent fluctuations in PT% and INR were noted during the hospital course, consistent with ongoing hepatic dysfunction. Fibrinogen levels remained within or above reference ranges, suggesting preservation of the acute-phase response.

Assessment of coagulation factors on Day 7 of admission, performed after administration of blood product transfusion, revealed normal or elevated levels of Factor V (153.2%, normal 70–120), Factor VIII (287.6%, normal 60–150), Factor IX (139.5%, normal 60–150), and Factor X (93%, normal 70–140), indicating preserved activity of several coagulation components despite abnormal prothrombin time. Factor VII was not measured. The preserved levels of vitamin K-dependent factors may indicate partial conservation of hepatic synthetic function despite acute hepatic injury and recent gastrointestinal blood loss.

On Day 7 of hospitalization, the patient developed clinical signs suggestive of severe hepatic dysfunction with prodromal features of hepatic encephalopathy. The patient was somnolent, fatigued, and adynamic, without perseveration, and exhibited sleep–wake cycle inversion. A mild flapping tremor was observed.

The patient was supported by medical and hemostatic management. Her clinical and biochemical status started to improve in the third week of admission. She was eventually discharged following a 23-day hospital stay. At discharge, supportive therapy, dietary measures, and outpatient follow-up were recommended.

## 3. Discussion

The hepatitis A virus (HAV) infection remains a major cause of acute viral hepatitis worldwide, despite pronounced regional differences in incidence and disease burden [1,8]. In childhood, HAV infection is typically considered a mild and self-limiting disease, with spontaneous recovery in the majority of cases [9,10]. However, recent epidemiological shifts toward intermediate endemicity have resulted in delayed exposure and increased disease severity in older children and adolescents, who may exhibit more complicated clinical courses [11].

Although severe complications are uncommon, HAV infection has been reported as a cause of severe hepatic dysfunction and, in rare cases, pediatric acute liver failure [12,13].

The present case illustrates a rare but clinically significant manifestation of acute hepatitis A, complicated by extreme hyperbilirubinemia, gastrointestinal bleeding, and transient neurological impairment.

Although the patient developed severe hepatic dysfunction with neurological manifestations, she did not fulfill the established diagnostic criteria for acute liver failure. The clinical course remained outside the diagnostic spectrum of acute liver failure and did not require liver transplantation or intensive liver failure management.

Coagulation abnormalities were observed in this patient during the course of acute hepatitis. Elevated factor VIII levels were detected; however, a comprehensive evaluation of the overall hemostatic balance was not performed. Therefore, the clinical significance of this finding in relation to the bleeding episode remains uncertain.

Gastrointestinal bleeding has only been sporadically reported as a complication of pediatric HAV infection and should not be attributed solely to coagulation abnormalities [14].

Although the presence of a duodenal ulcer in this patient raises the possibility that systemic stress associated with acute hepatitis A may have contributed to gastrointestinal bleeding, a direct causal relationship cannot be definitively established. Severe hepatic dysfunction may increase susceptibility to gastrointestinal mucosal injury; however, the coexistence of both conditions may also be incidental, and this limitation should be considered when interpreting the clinical findings.

## 4. Conclusions

A pediatric case of acute hepatitis A was presented, complicated by severe upper gastrointestinal bleeding, leading to the diagnosis of a previously unrecognized duodenal ulcer. The patient recovered with conservative medical management. Although a direct causal relationship cannot be established, the coexistence of acute hepatic dysfunction and gastrointestinal bleeding highlights the importance of careful monitoring for potentially life-threatening complications in children with hepatitis A.

## Figures and Tables

**Table 1:** Hematological parameters over time. Values outside the reference range are highlighted in bold.

Parameter	Day 1	Day 3	Day 4	Day 5	Day 7	Day 12	Day 23	Reference Values
HGB (g/L)	135	**76**	**66**	**73**	**65**	**106**	122	120–160
RBC (×10^12^/L)	4.56	**2.42**	**2.04**	**2.27**	**2.06**	**3.35**	3.63	3.8–5.3
HCT (L/L)	0.42	**0.21**	**0.19**	**0.22**	**0.19**	**0.32**	0.36	0.35–0.47
WBC (×10^9^/L)	**16.17**	**26.68**	**47.39**	**22.74**	**20.77**	9.19	11.39	4.5–11
PLT (×10^9^/L)	407	316	197	147	250	283	500	140–400

Abbreviations: HGB, hemoglobin; RBC, red blood cell count; HCT, hematocrit; WBC, white blood cell count; PLT, platelet count.

**Table 2:** Clinical and chemical parameters. Values outside the reference range are highlighted in bold.

Parameter	Day 1	Day 3	Day 4	Day 5	Day 7	Day 12	Day 23	Reference Values
Total bilirubin (µmol/L)	**194.3**	**216.4**	**458.6**	**772.1**	**1083.7**	**930.1**	**149.6**	<22
Direct bilirubin (µmol/L)	**117.8**	**125.5**	**376.1**	**837.1**	**744**	**793.1**	**55**	<3.4
AST (U/L)	**838**	**1530**	**929**	**885**	**1327**	**810**	**133**	35–40
ALT (U/L)	**1310**	**1214**	**590**	**605**	**843**	**608**	**132**	35–50
Albumin (g/L)	39	**27**	**30**	**30**	35	38	37	35–55
Total protein (g/L)	71.2	**43.3**	**47.5**	**43.2**	**49**	**56.5**	67.4	60–83
Amylase (U/L)	37	31	68	**216**	**309**	**188**	96	25–125
Lipase (U/L)	-	9	-	**196**	**199**	152	39	0–160

Abbreviations: AST, aspartate aminotransferase; ALT, alanine aminotransferase.

**Table 3:** Coagulation parameters during hospitalization (Days 1–23).

Parameter	Day 1	Day 3	Day 4	Day 5	Day 7	Day 12	Day 23	Reference Values
PT%	**62.5**	**60.2**	95.8	**72.9**	**57**	**54**	**77.4**	80–100%
INR	1.17	1.08	0.97	1.09	**1.22**	**1.25**	1.07	0.8–1.1
Fibrinogen (g/L)	2.52	3.43	2.55	2.97	**4.33**	**4.13**	3.71	2–4

## Data Availability

The data presented in this study are available upon request from the corresponding author. The data are not publicly available due to privacy and ethical reasons.
